# Validated dataset combining simulations and measurements for emission analysis of naturally ventilated dairy barns

**DOI:** 10.1016/j.dib.2025.112017

**Published:** 2025-09-01

**Authors:** Julian Hartje, Abu Zar Shafiullah

**Affiliations:** Thünen Institute for Agricultural Technology, Bundesallee 47, 38116 Braunschweig, Germany

**Keywords:** Computational fluid dynamics, Livestock building, Wind speed, Wind direction, Large opening, Ammonia

## Abstract

Quantifying emissions from naturally ventilated livestock buildings is challenging due to the large side wall openings. In addition, measurement campaigns are expensive and time consuming and are therefore limited to a few short measurement weeks during the year. However, emission factors or annual averages are extrapolated from these data sets. Simulations can complement this data set by extending it and thus broadening the basis for the extrapolation of emission factors or evaluation of the barn and management system. The dataset presented consists of solution data from computational fluid dynamics (CFD) simulations of naturally ventilated cattle barns and the corresponding simulation and geometry files. The simulations were validated using data sets from measurement campaigns in three naturally ventilated cattle barns in Germany. Together with weather data from the German Weather Service (DWD), weather situations that occurred outside the measurement weeks could be investigated. With the presented data set further investigations are possible. Together with the measured data, simulation techniques, data aggregation and the development of new numerical modelling approaches can be investigated in detail.

Specifications Table

**Table:** 

Subject	Computer Sciences
Specific subject area	Simulation data for evaluating and monitoring emissions in naturally ventilated dairy cattle barns
Type of data	Tables (.xlsx) and simulation files (.sim, .stl), figures and plots (.png), R-script (.txt) Processed
Data collection	Simulated data were generated using computational fluid dynamics (CFD), based on two sets of boundary conditions derived from: (i) measured data from the EmiDaT project, and (ii) meteorological data provided by the German Weather Service. All simulations were conducted using StarCCM+ (version 2020.3.1, Siemens PLM)
Data source location	Thünen Institute for Agricultural Technology Bundesallee 47 38116 Braunschweig, Germany
Data accessibility	Repository name: zenodo Data identification number: https://doi.org/10.5281/zenodo.16881506. Direct URL to data: https://zenodo.org/records/16881506.
Related research article	–

## 1 Value of the Data

• **Advancing simulation studies in livestock buildings:** the integration of numerical models with measurement data from real-world barn facilities enables in-depth investigations that yield valuable insights for a wide range of simulation studies related to naturally ventilated livestock buildings. This includes areas such as advanced turbulence modelling techniques, representation of animal heat and mass transfer, extended statistical analysis of measurement data and its influence on simulation accuracy, development of models to represent vegetation elements such as hedges and trees, including their impact on flow and emissions and enhanced modelling of structural features like adjustable curtains
• **Extending measurement campaigns through CFD simulations:** The linkage of empirical data with CFD simulations offers unique opportunities to extend the utility of measurement campaigns through validated numerical modeling, enabling improved assessments of emission dynamics in naturally ventilated barns and exploration of environmental conditions beyond the limited measurement periods, such as varying wind or temperature scenarios
• **Cross-platform CFD comparisons:** The defined boundary conditions used in the simulations support reproducibility and facilitate application in other CFD solvers, promoting transparency and comparative analysis across research efforts

## 2 Background

The data were generated within the context of a study that combined empirical measurements with computational fluid dynamics (CFD) simulations to better represent the emission behavior of dairy barns under varying environmental conditions. The dataset includes boundary condition inputs, simulation outputs, and the complete simulation and geometry files used in the modeling process.

Measuring emissions in naturally ventilated dairy barns presents challenges due to large sidewall openings, which makes it harder to determine the airflow. Additionally, measurement campaigns are resource-intensive and typically limited to short durations, which may not capture the full range of meteorological conditions relevant to annual emission estimates [[1], [2], [3], [4], [5], [6]]. This dataset was compiled in response to these limitations, with the aim of supplementing short-term measurements using simulation-based approaches.

It supports the associated research article by providing full access to the modeling framework, enabling reproducibility and offering a basis for further method development or comparison. The dataset is intended to assist other researchers in applying or adapting similar approaches in related contexts.

## 3 Data Description

The available data [7] consists of two main packages. Package 1 contains the simulations as sim-files, solution plots of these simulations as png-files stored in a zip-folder and the geometric models of the simulated barns as stl-files. Package 2 contains excel-files (Microsoft Excel (Version 365, Microsoft Corporation, Redmond, WA, USA)) with the results of the first simulations and the corresponding boundary conditions. As additional files, the used R-code (txt-file) and metadata with a short file description (pdf-file) have been added to the package.

**Fig. 1:**
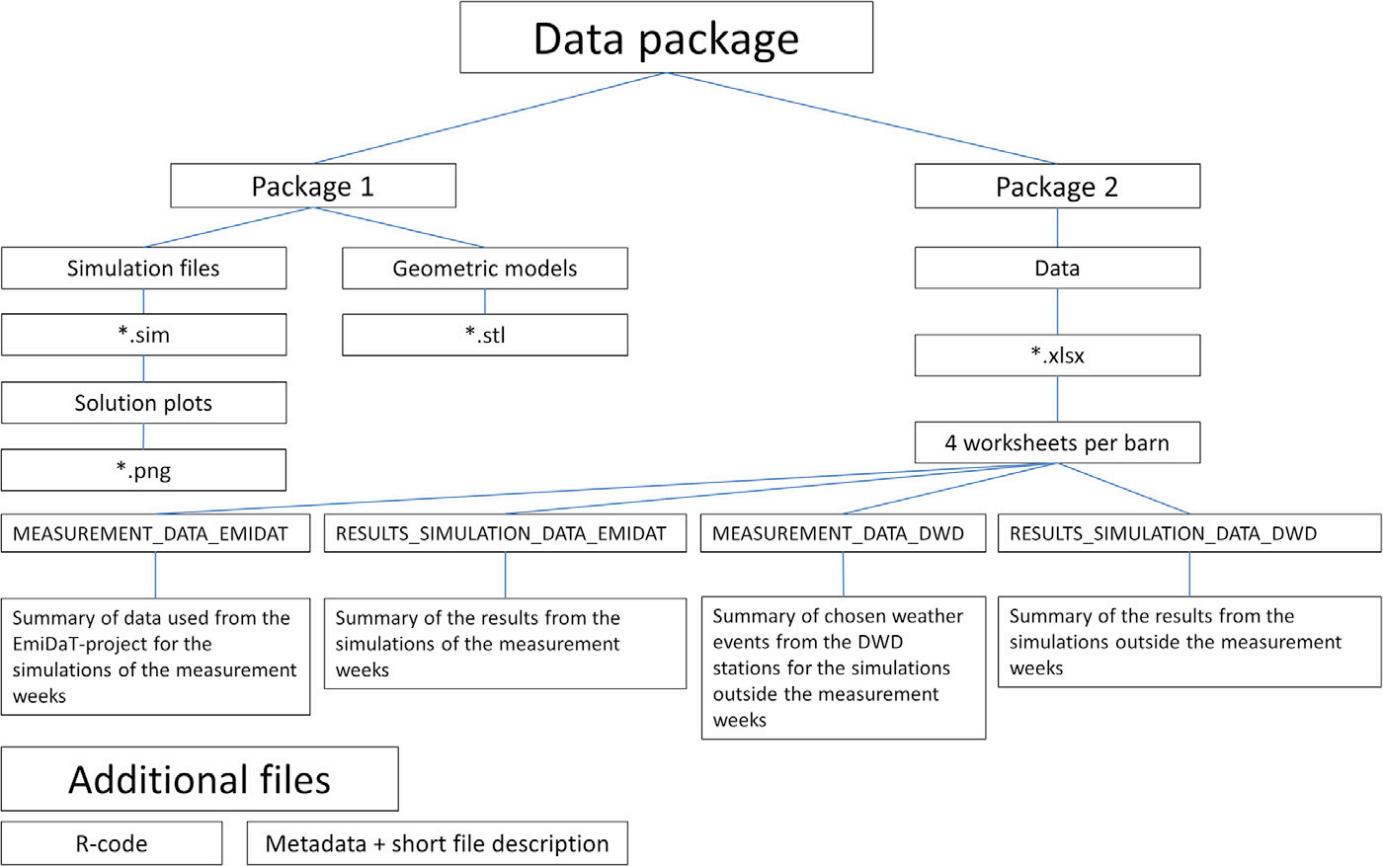
File-Structure of the data package.

An overview of the structure of the presented dataset is given in Fig. 1.

### 3.1 Simulation files

Due to storing capacities in the repository, only one simulation per barn is saved there. The other cases can be reproduced by changing the boundary conditions as explained in section “EXPERIMENTAL DESIGN, MATERIALS AND METHODS”.

**Fig. 2:**
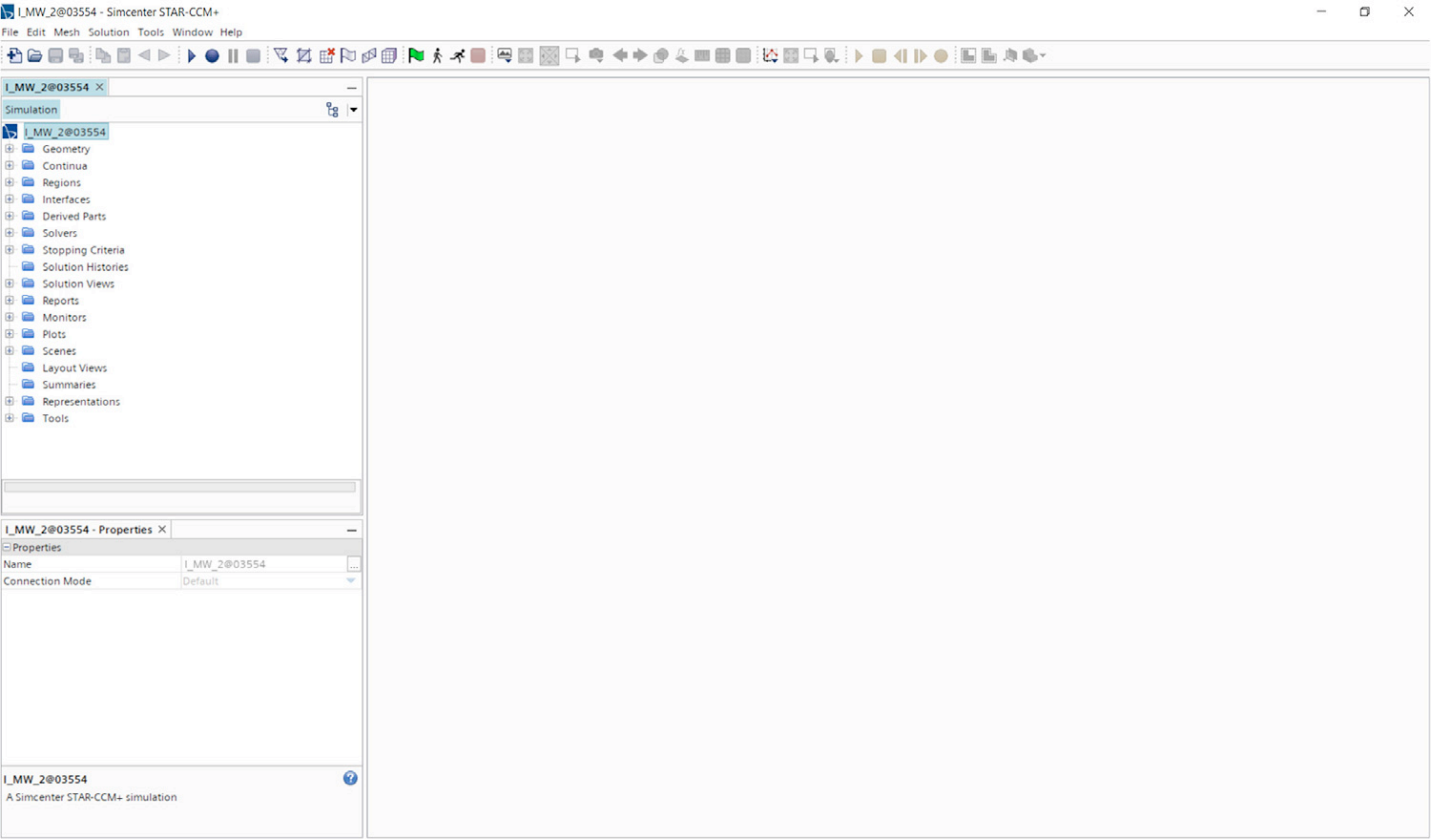
Snapshot of the GUI from StarCCM+.

All simulations were carried out using StarCCM+ version 2020.3.1 by Siemens PLM. They are stored in the software-specific file format *.sim and can be reused using the same software. StarCCM+ offers solutions for creating the geometric models, choosing solvers and numerical models for running the simulations as well as using a variety of tools for postprocessing the solution of the simulation. Fig. 2 shows a snapshot of the graphical user interface (GUI).

Table 1 gives an overview of the used models, boundary conditions, field functions and solution data and where they can be found within the software. Some parameters are stored in multiple places, i. e. the outside temperature. It serves as the reference temperature for all temperature related operations within the solver but also as the input temperature on the outer boundaries so that the incoming air has the correct temperature for the certain simulation. In these cases, multiple lines per variable indicate the multiple locations for them to be found.

**Table 1:** Overview of location of parameters and solution data in StarCCM+.

Models/boundary condition	Location in StarCCM+
All numerical models	Continua –> Physics 1 –> Models
Outside temperature	Continua –> Physics 1 –> Reference Values –> Reference temperature
	Continua –> Physics 1 –> Initial conditions –> Static temperature
	Regions –> air –> Boundaries –> all *velocity inlet* and *pressure outlet* boundaries –> Physics Values –> Static temperature
Wind direction	Tools –> Parameters –> inlet_angle
Wind speed	Tools –> Parameters –> *v*_10
NH_3_ background concentration	Continua –> Physics 1 –> Initial conditions –> Passive Scalar
	Regions –> air –> Boundaries –> all *velocity inlet* and *pressure outlet* boundaries –> Physics Values –> Passive scalar
NH_3_ surface concentration	Tools –> Field functions –> 38.NH3_surface_concentration
**Solution data**	
Volume flow through the barn	Plots –> sum_V Monitor Plot
Inside temperature	Plots –> temp_points –> Temp_point_inside Monitor
NH_3_ concentration inside the barn	Plots –> NH3_lines –> NH3_lines_inside_mean Monitor
Global normalized Residuals	Plots –> Residuals

The solution data from Table 1 is stored as plots in the file format *.png on the repository for every barn simulation and every case in a zip-folder called solution_plots.zip. The nomenclature for naming them follows the structure seen in Table 2. The different name-sections are separated by an underscore.

**Table 2:** Nomenclature for naming the solution plots of the simulations.

Barn identifier	Underlying dataset	Event number	Content
I II III	MW DWD	1 2 3 4 5 6	Residuals V NH3 T

MW = measuring week, DWD = German Weather Service, *V* = volume flow, NH3 = NH_3_-concentration inside the barn, T = temperature inside the barn (only barn I).

Thus, for example, the volume flow through barn I for event 4 of the measurement weeks can be seen in the file I_MW_4_V.png.

### 3.2 Geometric models

**Fig. 3:**
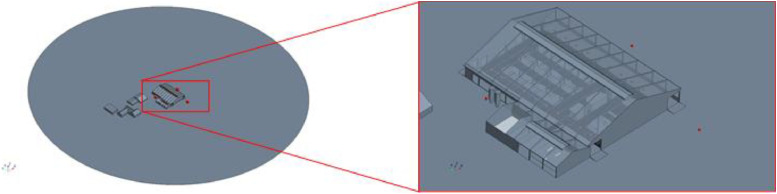
Geometric model of barn I.

**Fig. 4:**
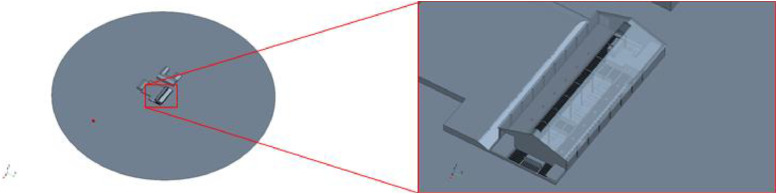
Geometric model of barn II.

**Fig. 5:**
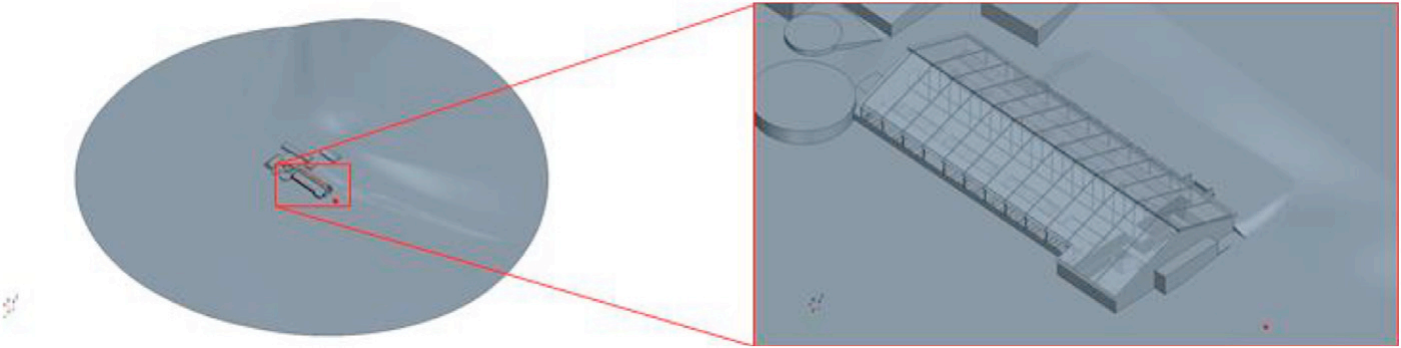
Geometric model of barn III.

The geometries for each barn are stored as *.stl files. They were created in the StarCCM+ CAD editor. The models were built using floor plans and own measurements on site. Not only the barns, but also the surrounding buildings and important features of the wider environment (mountain/valley) were taken into account. The model was constructed on a 1:1 scale. In order to make it easier to vary the wind direction at a later date, the entire simulation environment was constructed as a cylinder with the barn model on its base. Fig. 3, Fig. 4, Fig. 5 show the geometric models of the respective barns including surrounding buildings and a view of the simulation domain.

### 3.3 Data

An individual excel-file was created for each of the three barns. Each contains four worksheets, which are described in more detail below.

MEASUREMENT_DATA_EMIDAT

This worksheet contains an overview of the data used for the simulation from the EmiDaT project. The worksheet has 14 columns for barn I and 13 columns for barns II and III, as the temperature inside these barns was not measured. A detailed description is given in Table 3.

**Table 3:** Overview of column entries in the worksheet MEASUREMENT_DATA_EMIDAT.

Name of the column	Meaning of the content of the column
Measurement week	Indicates the measurement week in which the selected period is located
Period	Indicates the month of the year in order to obtain a classification in the season
Number of animals	Number of animals that were housed at this time
Averaging of the measurement data Start (UTC)	Start measured value for averaging over the selected period
Averaging of the measurement data Finish (UTC)	Final measured value for averaging over the selected period
Duration	Duration of the selected period in hours and minutes
number of measurement points	Number of available measured values within this period
wind direction in °	Averaged wind direction within this period and CoV of the data
windspeed in m·s^-1^	Averaged wind speed within this time period and CoV of the data
outside temperature in °C	Averaged ambient temperature within this period and CoV of the data
background concentration of NH_3_ outside the barn in mg·m^-3^ or ppm	Averaged background concentration within this period and CoV of the data
temperature inside the barn in °C	Averaged barn temperature within the period (only barn I) and CoV of the data
measured concentration of NH_3_ inside the barn in mg·m^-3^ or ppm	Averaged measured ammonia concentration within the period and CoV of the data
volume flow through the barn in m^3^·h^-1^	Averaged measured air volume flow through the barn within the period and CoV of the data

**Fig. 6:**
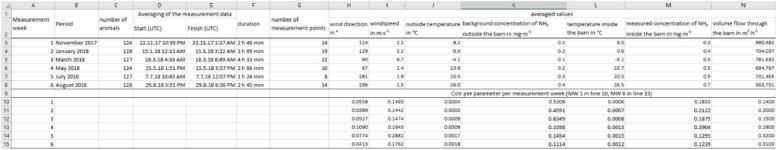
Snapshot of excel worksheet 1 for barn I.

Fig. 6 shows a snapshot of the presented data for barn I.

RESULTS_SIMULATION_DATA_EMIDAT

The averaged values from the previous worksheet were used as boundary conditions and target values (measured ammonia concentration in the barn). The resulting flow through the barn at the boundaries between the barn building and the surroundings was then evaluated. This worksheet provides an overview of these results. It has four columns for barn I and three columns for barn II and III. The columns are described in Table 4.

**Table 4:** Overview of column entries in the worksheet RESULTS_SIMULATION_DATA_EMIDAT.

Name of the column	Meaning of the content of the column
Measurement week	Data from the respective measurement week from the previous worksheet
Simulated temperature inside the barn in °C	Results of the barn temperature inside the barn at the same measuring points as in the real measurement
Simulated concentration of NH_3_ on the surfaces of the barn in ppm	Set NH_3_ concentration on the surfaces to achieve the concentration value at the measuring points from the previous worksheet in the simulation
Simulated volume flow through the barn in m^3^·h^-1^	Air volume flow through the barn determined in the simulation

MEASUREMENT_DATA_DWD

Similar to worksheet 1, the selected events outside the measurement weeks are listed here. The worksheet has nine columns. Table 5 gives an overview of the columns.

**Table 5:** Overview of column entries in the worksheet MEASUREMENT_DATA_DWD.

Name of the column	Meaning of the content of the column
Event	Numbering of the selected events
Period	Specifies the month of the year in order to obtain a classification in the season
ID of weather station	Indicates the ID of the weather station from the DWD network from which the data was recorded
Averaging of the measurement data Start (UTC)	Start measured value for averaging over the selected period
Averaging of the measurement data Finish (UTC)	Final measured value for averaging over the selected period
Duration	Duration of the selected time period in hours and minutes
wind direction in °	Averaged wind direction within this time period
windspeed in m·s^-1^	Averaged wind speed within this time period
outside temperature in °C	Averaged ambient temperature within this period

RESULTS_SIMULATION_DATA_DWD

The results of the simulations with the weather data from the DWD as boundary conditions are listed here. This worksheet has five columns for barn I and four columns for barns II and III. Table 6 lists the columns and their meaning.

**Table 6:** Overview of column entries in the worksheet RESULTS_SIMULATION_DATA_DWD.

Name of the column	Meaning of the content of the column
Event	Referencing the selected event from the DWD measurement data
simulated temperature inside the barn in °C	Results of the evaluation at the same measuring points as in the real measurement
simulated concentration of NH_3_ on the surfaces of the barn in ppm	NH_3_-concentration set on the surfaces to achieve the concentration value at the measurement points from the previous worksheet in the simulation
simulated NH_3_-concentration inside the barn in ppm	Resulting NH_3_-concentration inside the barn in the simulation at the real measuring points due to the flow situation and the set NH_3_-concentration on the surfaces
simulated volume flow through the barn in m^3^·h^-1^	Air volume flow through the barn determined in the simulation

### 3.4 R-Code

To investigate the relationships between environmental boundary conditions and simulated ammonia (NH₃) surface concentrations, structured datasets were created for each barn, containing variables such as temperature (T), wind speed (V), wind direction (D), and the modeled NH₃ concentration (y). A generic R function, *plot_relationships()*, facilitates exploratory analysis by visualizing both linear and nonlinear (quadratic) bivariate relationships between NH₃ and each predictor variable. For each predictor-response pair, scatterplots with fitted regression lines are generated alongside annotated R² values to quantify explanatory power.

Complementing these visual diagnostics, the function *extract_lm_uncertainty()* computes a comprehensive suite of uncertainty metrics from a fitted linear model. These metrics include coefficient standard errors, confidence intervals, p-values, and model-level statistics such as RMSE, R², adjusted R², and the F-statistic with its significance level.

**Fig. 7:**
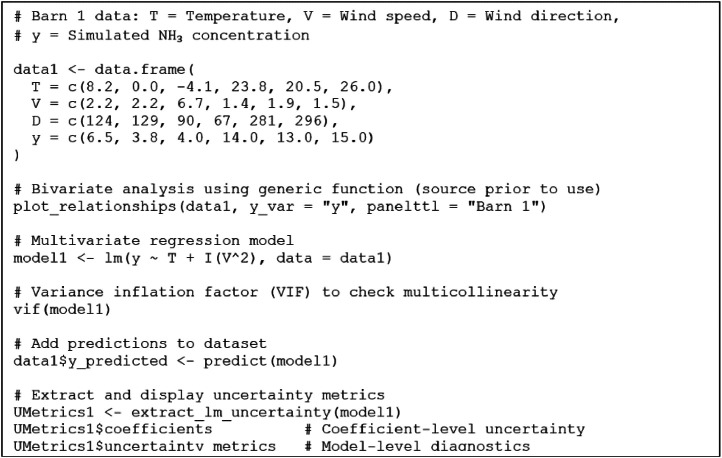
Extract of the R-script.

For illustration, after installing packages and sourcing the functions, the following R code block in Fig. 7 generates both graphical outputs of bivariate exploratory analyses and numerical summaries from multivariate regression models using the barn I dataset.

Prior to use, users should review the R script and install the required packages as specified in the accompanying file (*R scripts_Bivariate and Multivariate Analysis.txt*). The script is designed for ease of use, enabling implementation of the statistical analyses with minimal prior R experience by sequentially running code blocks in the R console.

## 4 Experimental Design, Materials and Methods

**Fig. 8:**
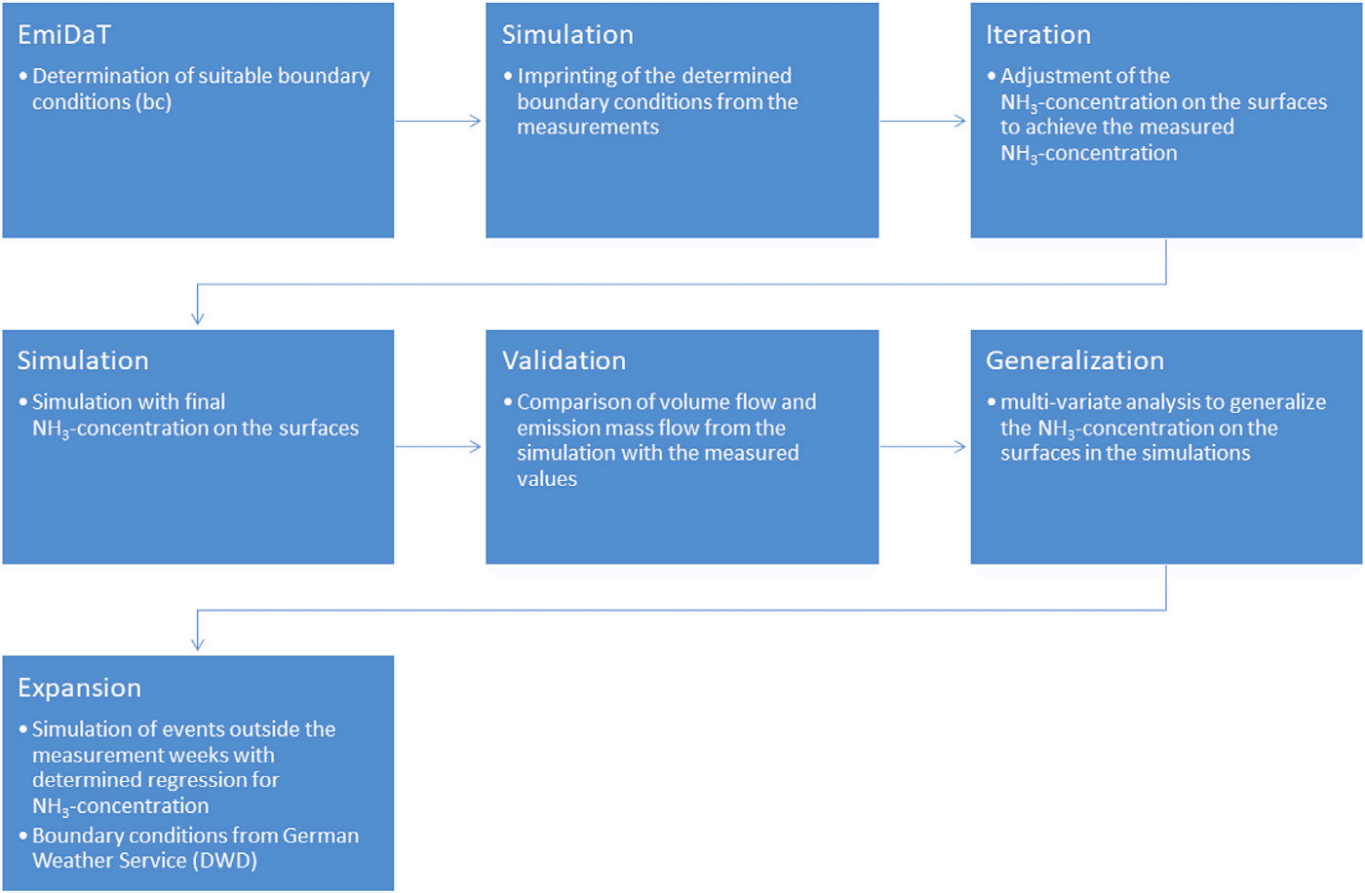
Path of data generation and further usage.

The generation and usage of data followed a path which can be seen in the following Fig. 8.

Each of these boxes is explained in detail in the following paragraphs.

### 4.1 EmiDaT

The basis of the data set used in this project are the measurement results of the project *“Determination of emission data for the assessment of the environmental impact of livestock housing” (EmiDaT)* [8]. In this project, different dairy barns were measured regarding the emission mass flow of ammonia and other parameters. The measurements were conducted for 6 measuring weeks starting in late 2017 and ending in late 2018. The measurements followed the VERA protocol [9]. The simulations were carried out for three of the investigated dairy barns. They differed in size, animal number and slurry management. Barn I housed an average of 126 cows and had a slurry cellar with slatted floor. Barn II was significantly smaller with 78 animals on average and an external slurry storage. In barn III, 199 cows were in there on average and the slurry on the solid floor was removed with a scraper. Barns II and III are located in the south-eastern part of Germany and barn I in the north-western area. A detailed description of the measurements conducted and the barn managements can be found in [8].

In order to later validate the numerical models with the measured results, the boundary conditions for these simulations had to be replicated in the simulation for the certain results to compare with. The parameters needed for the simulations as boundary conditions were the ambient temperature, the local wind speed and the local wind direction. The latter two were measured at a weather station in 10 m height near the barn. The temperature was measured close to the barn. The variables were usually measured at an interval of once per minute.

For each of the six measurement weeks for each barn, one event was extracted for simulation. Since the simulations are steady (i.e., time-independent), it was necessary to identify situations where the boundary conditions were as stable as possible, so the simulation would closely approximate reality. First, the evaluation was done visually by looking at the time series of three variables: ambient temperature, wind speed, and wind direction. Here, periods were picked that showed a small “visual variance” in at least two of the variables. These periods were evaluated further with two main tools. Box plots, generated with Microsoft Excel (Version 2021, Microsoft Corporation, Redmond, WA, USA), were used to visualize the variance of the three parameters across the periods. The interquartile range (Q3–Q1), mean, median, and data dispersion (whiskers) provided a good indication of the suitability as boundary conditions for the simulation. Furthermore, for quantitatively assessing the data consistency, the Coefficient of Variation (CoV≡ λ) was used, which is described by [3] and can be seen in Eq. (1) (Eq. (1)) It is the ratio of the root mean square deviation σ(k) and the mean $\bar{\phi }\left(k\right)$ for a variable k.

(1) $$CoV=\lambda \left(k\right)=\frac{\sigma \left(k\right)}{\bar{\phi }\left(k\right)},\text{where}\;\sigma \left(k\right)=\sqrt{\frac{1}{n}\sum_{j=k-n+1}^{k}{\left[\phi \left(j\right)-\bar{\phi }\left(j\right)\;\right]}^{2}},\bar{\phi }\left(k\right)=\frac{1}{n}\sum_{j=k-n+1}^{k}\phi \left(j\right)$$

This process led to the identification of one period (event) per measurement week per barn which results in 18 simulations for the validation set. The final value for each parameter is given as the mean over the chosen period. The CoV values are also presented in the excel-sheets for each barn.

**Fig. 9:**
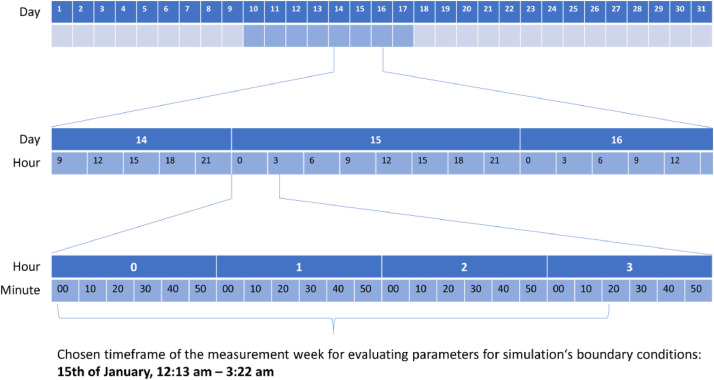
Exemplary visualization of determining the simulation event within measurement week 2 of barn I.

For example, Fig. 9 illustrates the selected timeframe of measurement data from week 2 in barn I.

### 4.2 Simulation I

**Fig. 10:**
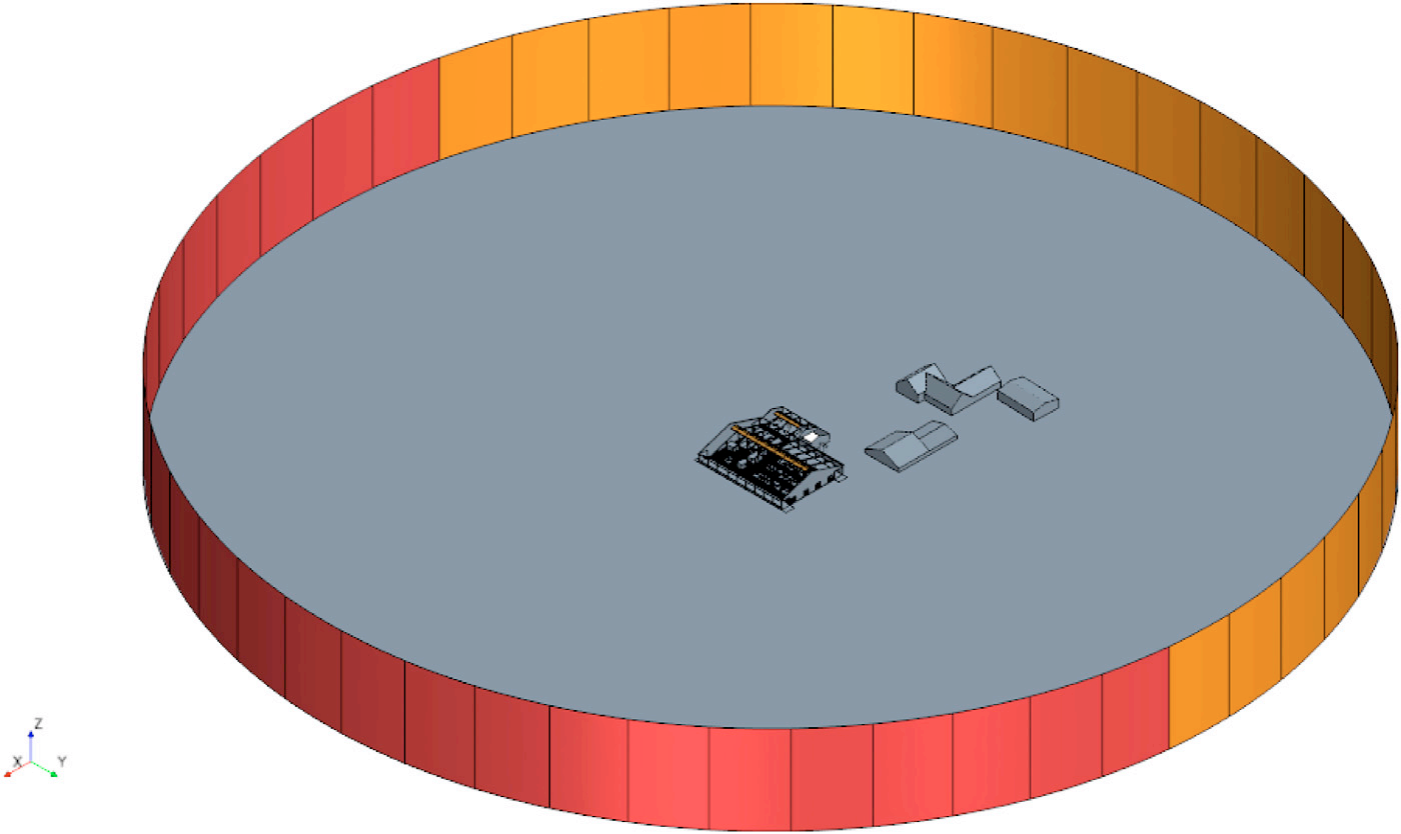
Visualization of the inlets (red) and outlets (orange) of the simulation domain for barn I.

The three-dimensional, steady-state simulations were carried out with *Simcenter StarCCM+* in version 2020.3.1 by Siemens PLM**.** The walls of the buildings had the no-slip boundary condition. The top boundary of the simulation domain was modelled as a slip-wall. The standard k-ε model was chosen for resolving the turbulence [10]. The animals were modelled as porous blocks using a single animal approach to eliminate the need for extensive pre-simulations [11]. Constant density and the Boussinesq approximation were further simulation settings due to the small thermal gradient between the ambient air and animal body temperature [12]. Inlets were modelled as velocity inlets and outlets as pressure outlets with 0 Pa pressure. Their positioning depends on the inlet angle. Fig. 10 shows the inlet and outlet distribution for barn I with the inlets being marked in red, and the outlets in orange.

Due to modelling the simulation domain as a cylinder, the outer surfaces can be divided into n equally sized parts. For barn I, *n* = 48, so that each inlet or outlet represents 7.5° of the entire lateral surface. For barns II and III, *n* = 24 was chosen, meaning each segment corresponds to a 15° section of the lateral surface. The simulations showed that for a better overall convergence, there should always be (n/2 + 1) inlets. When assigning the inlets and outlets to the surfaces accordingly, the inlet-section that contains the inlet angle, should be in the middle of the inlet chain. For example, if the inlet angle is α = 127°, the first velocity inlet to assign is on the surface which represents the angular section 120° ≤ α < 127.5° for barn I. From there, n/4 surfaces to both sides should be velocity inlets, while the remaining surfaces are designated as pressure outlets. In the simulation files, the surfaces are named according their smallest angular value they contain. For instance, for barn I, the section covering 22.5° ≤ α < 30.0° is labeled “22_5”.

The release of ammonia was not directly modelled (in a chemical way). It was imprinted on the surfaces (slurry surface, alleys, slatted floor) as a source for a passive scalar. The spatial resolution of this source was neglected and set to a constant value for all surfaces.

Convergence was considered achieved when the global residuals for continuity, momentum, energy, and turbulence dropped below $10^{-3}$. Additionally, the monitored values for ammonia concentration at the measurement points, volume flow through the barn, and climate parameters were required to show no significant variance.

With the identified boundary conditions from above and an initial ammonia surface concentration, the first simulation for each event was carried out and processed to the next step after meeting the convergence criteria.

### 4.3 Iteration and simulation II

In this step, the ammonia concentration at the simulated measurement points was first evaluated. To do this, the average was formed over all measurement points, analogous to the measurement procedure (see Eq. (2)).

(2) $${\bar{c}}_{NH_{3}}=\frac{1}{N}\sum_{i=1}^{N}c_{NH_{3},i}$$

If the ammonia concentration was too low, the surface concentration of ammonia in the simulation was manually increased. If it was too high, it was reduced accordingly. The simulation was then restarted and allowed to run until the ammonia concentration converged again. The described comparison between simulated and measured ammonia concentrations was then repeated. This iterative process continued until the values in the simulation equalled the measured concentration. The adjusted ammonia concentration from the simulation is an important outcome variable, used in the statistical analysis as outlined in the R-Code and Generalization sections of this paper.

### 4.4 Validation

The simulations were then validated by comparing the resulting volume flow and emission mass flow in the simulation with the measured values of the EmiDaT project.

#### 4.4.1 Evaluation of volume flow

To get the volume flow through the barn $\overset{˙}{V}$ in m³·s^-1^, the area of the sidewall openings A_i_ in m² of the barn and the velocity component $v_{⊥}$ perpendicular to that area in m·s^-1^ need to be evaluated. This was done via field functions in the solver for each element on the openings. The resulting volume flow for the barn is calculated via the sum over all elements in the openings generally following Eq. (3).

(3) $${\overset{˙}{V}}_{barn}=\sum_{i=1}^{N}v_{⊥,i}\cdot A_{i}$$

**Fig. 11:**
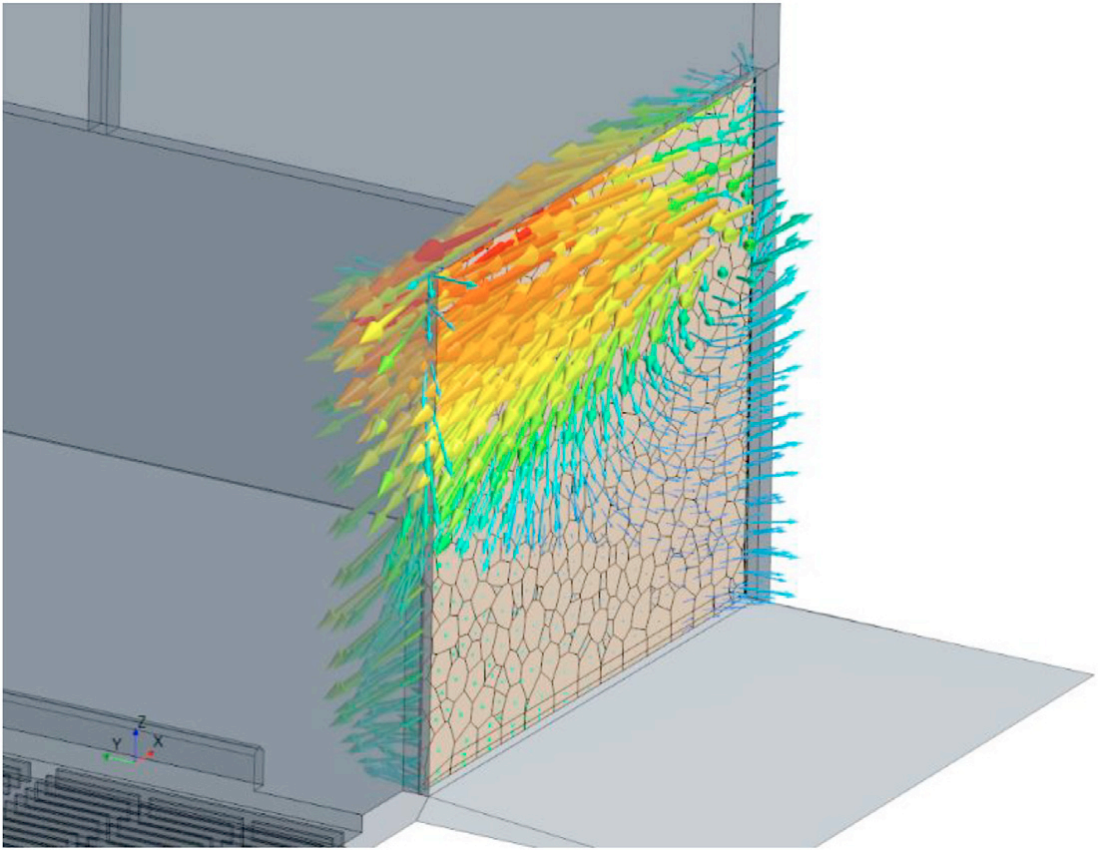
Examplary visualization of flow through a sidewall opening of a barn as a vector plot.

In detail, this was evaluated for each coordination direction. Fig. 11 shows an exemplary visualization of a flow through a sidewall opening of a barn. It can be seen that some areas of the opening function as inlets where the air is moving into the barn and some as outlets where the air is coming out of the barn at the same time. For the volume flow through the barn, only the part of the flow that points outward of the barn is considered as there are no sources of flow inside the barn.

In order to evaluate the volume flow coming out of the barn, the area of elements with a velocity vector pointing outwards and the velocity component perpendicular to that surface are needed. As the barn is built up perpendicular to the coordinate axis, the two parameters can be directly addressed via field functions. In the given example, the area is detected depending on the velocity component in negative y-direction via an if condition. The syntax of StarCCM+ follows the scheme “condition ? if true: if not true”. For the certain case, this results in “$Velocity[1] < 0 ? mag(${Area}): 0” (if the velocity in y-direction is smaller than zero, return the area of that element and if not, return zero). In the same way, the velocity itself is evaluated: “$Velocity[1] < 0 ? abs($Velocity[1]) : 0”. It is important to use the absolute value here as the volume flow through each element should add up to the final volume flow of the barn. Finally, the product of the two field functions returns a positive value for vectors having a velocity component in negative y-direction and 0 for the other elements. This evaluation is supposed to be done for every direction and every sidewall opening of the barn.

#### 4.4.2 Estimation of ammonia emission mass flow

The product of the volume flow through the barn $\overset{˙}{V}$ in m³·s^-1^ and the measured concentration of ammonia c in g·m^-3^ gives the emission mass flow $\overset{˙}{m}$ (Eq. (4)) in g·s^-1^. The ammonia concentration c is determined as an average over all measuring points [8].

(4) $${\overset{˙}{m}}_{NH_{3}}={\overset{˙}{V}}_{barn}\cdot {\bar{c}}_{NH_{3}}$$

#### 4.4.3 Evaluation of model performance

For validating the simulation, an appropriate measure is required. In our case, the Normalized Mean Square Error (NMSE) was chosen [13]. It is defined in Eq. (5) as follows:

(5) $$NMSE=\frac{1}{N}\sum_{i=1}^{N}\frac{{\left(P_{i}-M_{i}\right)}^{2}}{\bar{P}\cdot \bar{M}}.$$

Here, P_i_ and M_i_ are the predicted and measured values from the $i^{th}$ sample (i=1,2,…,N) respectively, and $\bar{P}$ and $\bar{M}$ are their arithmetic means. With this definition, the closer the NMSE gets to zero, the better is the agreement. The investigations were done for the measured and simulated volume flow through the barn. For further evaluations, box plots were also used for illustrating the variance between the simulation results and the measured values.

### 4.5 Generalization

To elucidate generalized relationships between these climatic variables and ammonia surface concentrations, we conducted both bivariate and multivariate regression analyses using R (version 4.4.2) [14]. The bivariate analyses separately examined linear and nonlinear effects of temperature, wind speed, and wind direction, while the multivariate regression assessed their combined predictive capabilities and potential interactions.

Ensuring full transparency and reproducibility, all related R scripts are publicly accessible via the repository [7]. These scripts enable users to:

• Reproduce the complete statistical analyses across all barns, facilitating validation and comparative studies;
• Explore individual and combined effects of meteorological variables on ammonia concentrations through straightforward functions;
• Compute detailed uncertainty metrics at the coefficient level, including confidence intervals, standard errors, and p-values;
• Obtain comprehensive model diagnostics such as RMSE, R-squared, adjusted R-squared, F-statistics, and variance inflation factors (VIF) for assessing model fit and multicollinearity.

By providing these resources, we enhance the transparency, usability, and adaptability of our dataset and statistical analysis. Researchers with minimal R experience can implement the analyses by sequentially running annotated code blocks, making this workflow broadly accessible. Moreover, the modularity of the scripts facilitates easy extension or adaptation to new datasets, barns, or environmental conditions, thereby supporting ongoing scientific validation and broader methodological generalization.

### 4.6 Expansion

In order to simulate events which took place outside the measurement periods, the two main values needed are again the boundary conditions (temperature, wind speed, wind direction) and the ammonia concentration on the surfaces for the simulation. The latter one was derived via the analysis in the generalization step. The climatic boundary conditions were derived using climate data from a nearby weather station of the German Weather Service. The values for the required parameters are publicly accessible and stored as hourly averages. When selecting the new boundary conditions, care was taken to choose those that differ from the previously simulated ones. Simulations were then carried out with these values following the same setup as described in section Simulation I. The results regarding emission mass flow can be compared to the other simulation results. As a further comparison measure, the literature value for the emission behavior provided by the German technical instruction on air quality control (TA-Luft) [15] was considered as well.

## Limitations

The simulation files including the solution (.sim) can only be opened with StarCCM+. If the files are loaded into a different solver a different file type is required. An attempt can be made to provide this data type after consultation with the corresponding author.

The geometric models of the barns presented here are specific to the barns investigated in the EmiDaT project.

## Ethics Statement

The authors have read and follow the ethical requirements for publication in Data in Brief. We confirm that our work does not involve human subjects, animal experiments, or any data collected from social media platforms.

## Credit Author Statement

**Julian Hartje**: Conceptualization, Data Curation, Formal analysis, Writing – original draft. **Abu Zar Shafiullah**: Data curation, Formal analysis, Visualization, Writing – review and editing.

## Declaration of Generative AI and AI-Assisted Technologies in the Writing Process

During the preparation of this work the authors used ChatGPT and DeepL in order to improve the written language. After using these tools, the authors reviewed and edited the content as needed and take full responsibility for the content of the published article.

## Data Availability

ZenodoApplication of digital twins to extend the emission data of naturally ventilated dairy cattle barns (Original data).
